# Discovery of Antibiofilm Activity of Elasnin against Marine Biofilms and Its Application in the Marine Antifouling Coatings

**DOI:** 10.3390/md19010019

**Published:** 2021-01-05

**Authors:** Lexin Long, Ruojun Wang, Ho Yin Chiang, Wei Ding, Yong-Xin Li, Feng Chen, Pei-Yuan Qian

**Affiliations:** 1 SZU-HKUST Joint PhD Program in Marine Environmental Science, Shenzhen University, Shenzhen 518000, China; llongaa@connect.ust.hk; 2 Department of Ocean Science and Hong Kong Branch of Southern Marine Science and Engineering Guangdong Laboratory, Guangzhou, The Hong Kong University of Science and Technology, Clear Water Bay, Kowloon, Hong Kong 999077, China; rwangaw@connect.ust.hk (R.W.); hychiang@connect.ust.hk (H.Y.C.); 3 Colleague of Marine Life Science, Ocean University of China, 5 Yushan Road, Qingdao 266100, China; dingwei@ouc.edu.cn; 4 Department of Chemistry, The University of Hong Kong, Pokfulam, Hong Kong 999077, China; 5 Institute for Advanced Study, Shenzhen University, Shenzhen 518060, China

**Keywords:** elasnin, biofilms, marine, biofouling, natural products

## Abstract

Biofilms are surface-attached multicellular communities that play critical roles in inducing biofouling and biocorrosion in the marine environment. Given the serious economic losses and problems caused by biofouling and biocorrosion, effective biofilm control strategies are highly sought after. In a screening program of antibiofilm compounds against marine biofilms, we discovered the potent biofilm inhibitory activity of elasnin. Elasnin effectively inhibited the biofilm formation of seven strains of bacteria isolated from marine biofilms. With high productivity, elasnin-based coatings were prepared in an easy and cost-effective way, which exhibited great performance in inhibiting the formation of multi-species biofilms and the attachment of large biofouling organisms in the marine environment. The 16S amplicon analysis and anti-larvae assay revealed that elasnin could prevent biofouling by the indirect impact of changed microbial composition of biofilms and direct inhibitory effect on larval settlement with low toxic effects. These findings indicated the potential application of elasnin in biofilm and biofouling control in the marine environment.

## 1. Introduction

A biofilm is a microbial community attached to a surface [1]. It consists of microbial cells massed in the matrix of extracellular polymeric substances, which contain a large variety of biopolymers such as proteins, nucleic acids, lipids, and other substances [2]. Biofilms can be made up of a single microbial species or multiple species that colonize biotic or abiotic surfaces [3,4]. The elaborate biofilm architecture protects the microbes in biofilms and provides spatial proximity and internal homeostasis needed for growth and differentiation [3,4,5]. This composition makes microbial cells more resistant than their planktonic counterparts to diverse external insults such as antimicrobial treatment, poisons, protozoans, and host immunity [6,7]. For example, biofilms can render organisms 10- to 1000-fold less susceptible to antimicrobial agents; furthermore, organisms in multi-species biofilms are less susceptible to antimicrobial treatment than those in mono-species biofilms because of their complex interactions [6,8,9].

In the marine environment, biotic and abiotic surfaces are rapidly colonized by microorganisms and subsequent biofilm formation composed of bacteria, diatoms, fungi, unicellular algae, and protozoa [5,10], which creates a big problem for humans. Marine biofilms are critical in inducing biofouling and biocorrosion in the marine environment. Biofilms are important to habitat selection and settlement of many sessile marine organisms; for example, invertebrate larvae can distinguish biofilms composed of different microbial community structures to settle on or not [11,12,13]. The metabolites produced by microorganisms such as hydrogen sulfide, various acids, and ammonia destroy various materials. Every year, biofouling and biocorrosion contribute to enormous economic losses worldwide in industries, including heat exchange, oil and gas processing, storage and transportation, and drinking and wastewater industries [14,15,16]. Moreover, the persistence and transmission of harmful or pathogenic microorganisms and their genetic determinants within the marine biofilms pose a great threat to human beings [5].

Given the continuous increase in economic loss and the potential threats caused by the formation of marine biofilms, effective and economic control methods are necessary. At present, antibiofilm/antifouling coatings are the widely used and are an easy control method in the marine environment. However, the chemical inhibitors used in traditional coatings, such as amines, amides, organic tin, and cuprous oxide, are toxic and harmful, have no favorable environmental profile, and can be bioaccumulated. Increasing attention is focused on the use of natural products to develop effective and less hazardous coatings. Natural compounds with antibiofilm activities generated from the metabolic mechanism of microorganisms can be an ideal substitute for the traditional chemical biocides, presenting environmentally friendly properties such as low toxicity and biodegradability. However, insufficient productivity and difficulty in synthesis limit the development of naturally synthesized compounds [14,16,17].

Among the marine biofilms, as the early colonizers, bacteria are an important factor in determining the structure and function of a mature biofilm [5,14,18]. Therefore, bacteria from marine biofilms can be great targets for the discovery of antibiofilm compounds. In the present study, we used bacteria isolated from marine biofilms to screen for antibiofilm compounds, which led to the discovery of the potent antibiofilm activity of elasnin. With high productivity, elasnin-based coatings were consequently prepared, and their activities against natural multispecies marine biofilms were assessed in the field test.

## 2. Results

### 2.1. Isolation and Identification of Biofilm Inhibition Compounds

**Figure 1:**
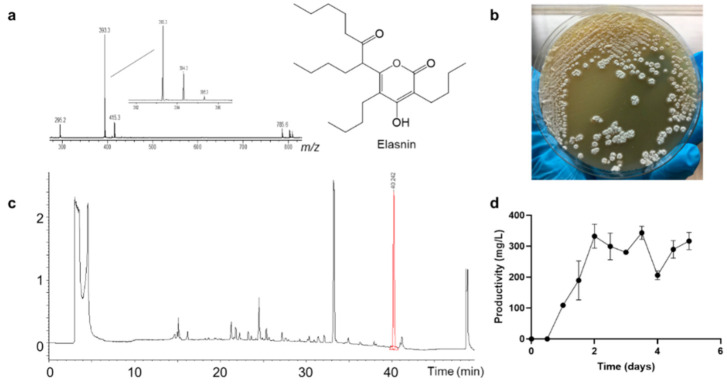
Elasnin was produced by *Streptomyces mobaraensis* DSM 40847 with high yield. (**a**) Mass spectra (ESI) and structure of elasnin; (**b**) growth of *S. mobaraensis* DSM 40847 on the GYM (Table S1) agar plate; (**c**) high-performance liquid chromatography (HPLC) analysis of the crude extracts of *S. mobaraensis* DSM 40847; (**d**) time course of the production of elasnin in AM4 medium at 30 °C.

During our screening project, secondary metabolites produced by *Streptomyces mobaraensis* DSM 40847 (Figure 1b) exhibited strong biofilm inhibition activity against marine bacteria *Staphylococcus aureus* B04. Fractionation coupled with biofilm inhibition assay led to the identification of the main bioactive compound fraction 16 (Figure S1), which was produced at a high yield (approximately 332 mg/L), thereby achieving the maximum productivity after 2 days of incubation. Fraction 16 was subsequently purified using high-performance liquid chromatography (HPLC) and structurally characterized as a known compound elasnin using ultra-performance liquid chromatography-tandem mass spectrometry (UPLC–MS/MS) and nuclear magnetic resonance (NMR) spectroscopy (Figure 1, Supplementary Materials Figures S2 and S6 and Table S2).

Fraction 16: Appearance: colorless, viscous oil; UV (λmax): 291 nm; ^1^H NMR (500 MHz, DMSO-*d*_6_):δ0.80 (t, 3H), δ0.83 (t, 3H), δ0.86 (t, 3H), δ0.90 (t, 3H), 1.04~1.45 (overlapped, 18H), 1.61 (m, H, H_a_-13), 1.86 (m, H, H_b_-13), 2.32~2.47 (m, 6H), 3.87 (dd, 1H, *J* = 8.8, 5.7 Hz, H-6), 10.49 (s, 1H, 3-OH); ^13^C NMR (150 MHz, DMSO-*d*_6_): δ13.8, 13.8, 13.8, 13.8, 22.0, 22.1, 22.1, 22.1, 22.8, 22.8, 23.9, 27.6 (C-13), 28.9, 30.0, 30.5, 31.5, 39.8 (C-8), 52.8 (C-6), 103.1 (C-2), 114.2 (C-4), 154.5 (C-5), 163.6 (C-3), 163.8 (C-1), 206.8 (C-7); LC-ESI-MS: (*m/z*) [M + H]^+^ 393.3.

### 2.2. Elasnin Could Inhibit the Biofilm Formation of Multiple Strains of Bacteria Isolated from Marine Biofilms

**Figure 2:**
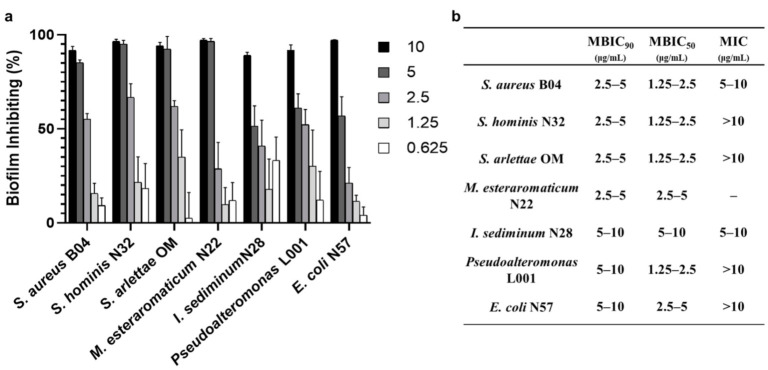
Antibiofilm activities of elasnin against bacteria isolated from marine biofilms. (**a**) Minimum concentration needed for inhibiting biofilm formation; (**b**) summary of minimum biofilm inhibitory concentration (MBICs) and minimal inhibitory concentration (MICs) of elasnin against test strains.

Ten strains isolated from marine biofilms—*Vibrio alginolyticus* B1, *Erythrobacter* sp. HKB8, *Ruegeria* B32, *S. aureus* B04, *S. hominis* N32, *S. arlettae* OM, *Microbacterium esteraromaticum* N22, *Idiomarina sediminum* N28, *Pseudoalteromonas* L001, and *Escherichia coli* N57—were used as targets in the minimum biofilm inhibitory concentration (MBIC) assay and minimal inhibitory concentration (MIC) assay. The MBIC is the lowest concentration of a compound that resulted in a certain reduction in the attached cells while the MIC is the lowest concentration needed for inhibiting the visible growth of planktonic cells. Among the ten strains, seven successfully formed biofilms during the test, and three (*V. alginolyticus* B1, *Erythrobacter* sp. HKB8, and *Ruegeria* B32) cannot form biofilms under the testing situation. For the seven biofilm-forming strains, the biofilms of four Gram-positive strains were sensitive to elasnin treatment with MBIC_90_ of 2.5 to 5 μg/mL and MBIC_50_ of 1.25 to 5 μg/mL, whereas the MBIC_90_ and MBIC_50_ of elasnin against Gram-negative strains ranged from 5 to 10 μg/mL and 1.25 to 10 μg/mL, respectively (Figure 2a). The MICs of nine strains were determined except for *Microbacterium esteraromaticum* N22 due to its inability to grow under the testing conditions. Elasnin inhibited the planktonic cells of *S. aureus* B04 and *Idiomarina sediminum* N28 from growing with MICs of 5 to 10 μg/mL while for other strains the MICs of elasnin were above 10 μg/mL (Figure 2b). Overall, comparing to antimicrobial activity, elasnin shows more significant efficiency in inhibiting biofilm formation.

### 2.3. Preparation of Elasnin-Based Antibiofilm Coatings

Given the high antibiofilm efficiency and high productivity, elasnin-based antibiofilm coatings were prepared and immersed in a fish farm to evaluate their efficiency against natural marine biofilms. In the present study, we used a crude extract of *S. mobaraensis* DSM40847 that contained high concentrations of elasnin (=336.64 mg/L in n-hexane, Figure S7) instead of pure elasnin. The extracts were mixed with polyurethane (polymer) on the basis of poly ε-caprolactone and applied directly on the surface of glass slides. The concentrations of the coatings were calculated on the basis of the percentage of crude extracts in total coatings (polymer and crude extracts) by weight. As such, other compounds in low amounts in the fractionated extract might affect the results of our field testing. However, their effect should be negligible because we did not detect any significant effects of the minor compounds on the crude extracts (Figure S7) and the crude extracts has the same biofilm inhibition efficiency as elasnin shows (MBIC of 0.8–4 and 4–20 μg/mL to *S. aureus* B04 and *E. coli* N57, respectively).

### 2.4. Release Rate of Elasnin from Antibiofilm Coatings

**Figure 3:**
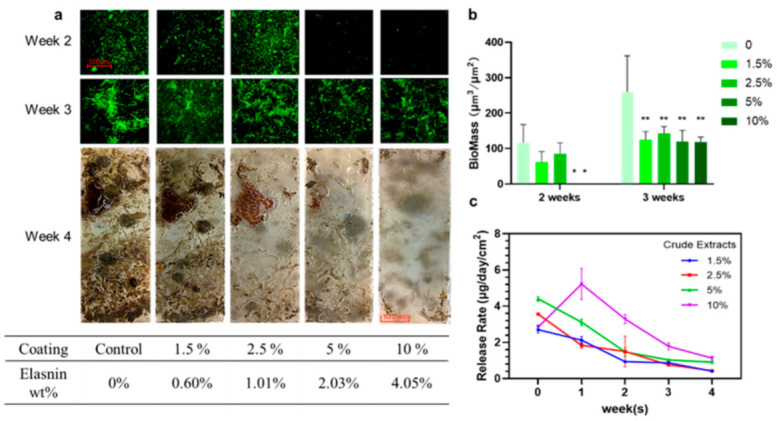
Antibiofilm (weeks 2 and 3) and antifouling (week 4) performance of elasnin-based antibiofilm coatings. (**a**) Confocal laser scanning microscopy (CLSM) images (weeks 2 and 3) of the coated surface (week 4); (**b**) Biomass of biofilms observed by CLSM. (**c**) Monitoring of the release rate of elasnin into the artificial seawater; Biomass is calculated using Comstat 2.1 on the basis of the CLSM images and values that are significantly different among elasnin-based antibiofilm coatings, and the control groups are indicated by asterisks: * for *p* < 0.05 and ** for *p* < 0.01.

The release rate of elasnin from the coatings was dependent on time and concentration during the 4 weeks observation (Figure 3c). In general, the release of elasnin was maintained at a low rate throughout the period; the higher the concentration, the faster the release of elasnin into the artificial seawater. The highest release rate of approximately 5 μg day^−1^ cm^−2^ occurred in the second week for the concentration of 10 wt%; for other concentrations, the maximum release rate was approximately 4 μg day^−1^ cm^−2^ in the first week. The release rate decreased over time and depended on the total amount of elasnin remaining in the coatings. After immersion for 4 weeks, the release rate dropped to approximately 1 μg day^−1^ cm^−2^ for the concentrations of 10 wt% and 5 wt% and 0.5 μg day^−1^ cm^−2^ for 1.5 wt% and 2.5 wt%.

### 2.5. Elasnin-Based Coatings Inhibited the Formation of Multi-Species Biofilms and the Attachment of Large Biofouling Organisms in the Marine Environment

The performance of the antibiofilm coatings was assayed every week from the second to the fourth week by direct and confocal laser scanning microscopy (CLSM) observation (Figure 3a). Based on the quantitative analysis of CLSM images, the average biofilm biomass on the slides without elasnin was 116.44 μm^3^ μm^−2^ in the second week and 259.95 μm^3^ μm^−2^ in the third week, whereas the average biomass of biofilms measured on 5 wt% and 10 wt% coating slides was less than 0.1 μm^3^ μm^−2^ in the second week and less than 120 μm^3^ μm^−2^ in the third week. For coatings with low concentrations (1.5 wt% and 2.5 wt%), no significant differences were observed with regard to average biomass (61.97 and 84.73 μm^3^ μm^−2^, respectively) in the second week, but the biomass was significantly lower than that in the control (259.95 μm^3^ μm^−2^) in the third week, with an average biomass of approximately 125 and 145 μm^3^ μm^−2^, respectively (Figure 3b). In the fourth week, slides coated with low concentrations of elasnin (1.5 wt%, 2.5 wt%, and control) were fouled by large marine organisms, whereas those coated with high concentrations of elasnin exhibited an anti-macrofouling activity and almost no larval settlement, except for a small area near the edges because of the edge effects commonly found on testing panels. Elasnin-based antibiofilm coatings inhibited the biofilm formation of multiple bacterial species in the first 2 weeks. However, after immersion for 4 weeks, the glass slides coated with low concentrations of elasnin were eventually covered with large biofouling organisms probably because of the low releasing rate of the elasnin after 3 weeks.

### 2.6. Elasnin Changed the Microbial Community Structure of Natural Marine Biofilms

**Figure 4:**
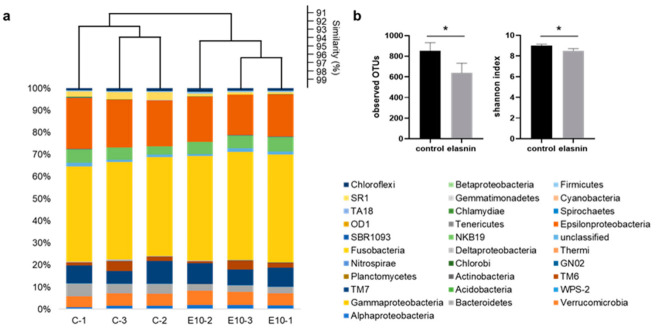
Composition analysis of biofilms grown on the control slides (without crude extracts) and treatment slides (with 10% CR coatings). (**a**) Similarity comparison of microbial compositions among biofilms on control slides (C-1,2,3) and 10 wt% elasnin-based coatings (E10-1,2,3) based on the beta-diversity (Bray–Curtis) at the phylum level. (**b**) Alpha-diversity of biofilms at the phylum level. The difference between the two types of biofilms is calculated using Student’s t-test and is indicated by an asterisk: * for *p* < 0.05.

Considering that the number of biofilms developed by the end of the second week was limited, and macrofoulers had overgrown by the end of the fourth week, only the 3 week-old biofilms developed on 10 wt% coatings and those on the control glass slides (coated with poly ε-caprolactone-based polyurethane only) were selected for 16S amplicon analysis to determine the changes in biofilm microbial community triggered by elasnin. A total of 3,000,000 16S rRNA gene sequences (500,000 per sample) were classified into 31 phyla (*Proteobacteria* were classified down to the class level). The microbial composition of the biofilms differed between the 10 wt% coatings and the control slides, as confirmed by alpha- and beta-diversity analysis. In the Bray–Curtis dissimilarity (beta-diversity) dendrogram (Figure 4a), the control and treatment groups were clustered separately on the basis of the differences in microbial abundance among the samples; the observed operational taxonomic units (OTUs) and Shannon diversity for the treated biofilm were significantly lower than those in the control group (Figure 4b), indicating that species richness and diversity in the treated biofilms were reduced.

### 2.7. Elasnin Inhibited the Larval Settlement of Balanus Amphitrite with a Low Toxic Effect

**Figure 5:**
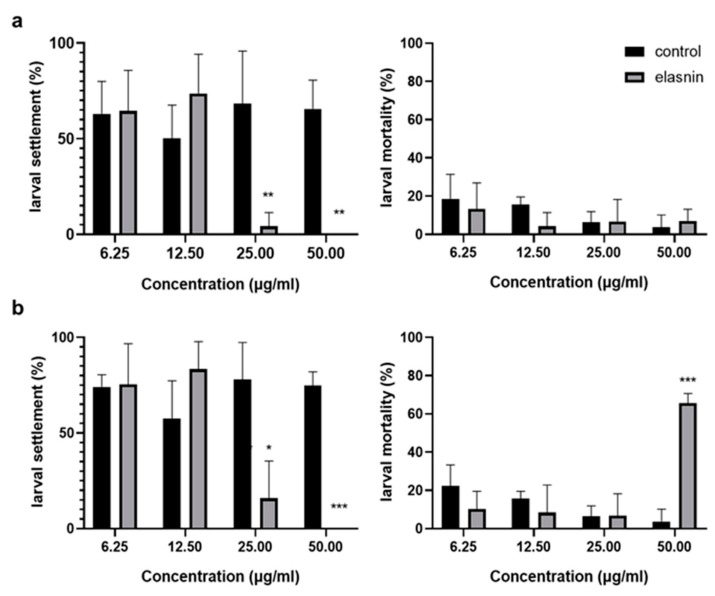
Effect of elasnin on the percentage of larval settlement and mortality rate of *B. amphitrite* after treatment for (**a**) 24 h and (**b**) 48 h. The differences between the control and treatment groups are calculated using Student’s t-test and are indicated by asterisks: * for *p* < 0.05, ** for *p* < 0.01, and *** for *p* < 0.001.

The antilarval settlement activity of elasnin was measured using *B. amphitrite*, and its possible toxic effects were preliminarily assessed by the mortality rate. When the larvae were exposed to elasnin for 24 h, the larval settlement was significantly inhibited at concentrations above 12.5 μg/mL. No increase in mortality rate was observed at a concentration range from 6.25 to 50 μg/mL compared with the control groups (Figure 5a). After 48 h exposure to elasnin, the settlement inhibition was slightly reduced for the concentration of 25 μg/mL, whereas for the concentration of 50 μg/mL, the inhibitory effect was significant. An increased mortality rate of around 65% and reduced vitality of larva were observed at concentrations of 50 μg/mL after 48 h exposure, but no significant changes were exhibited in the mortality rate for other concentrations of the group compared with the control (Figure 5b).

## 3. Discussion

Here, we used bacteria isolated from marine biofilms to screen for the antibiofilm compounds that targeted the marine biofilms, which led to the discovery of the antibiofilm activity of elasnin. Elasnin-based coatings were then prepared with simple and cost-effective methods, and its efficiency against multi-species biofilms was tested in the natural marine environment.

The predictive validity of the screening assay is an important determinant of the success of drug discovery [19,20]. Marine biofilms are complex mixed-species microbial communities with specific intraspecies and interspecies communication and interaction [21,22]. Therefore, the simulation of the marine biofilm formation is difficult under laboratory conditions. In accelerating and simplifying the screening for antibiofilm compounds against marine biofilms with high predictive validity, bacteria isolated from marine biofilms were selected as the target for the bioassay. Consequently, the potential inhibitory activity of elasnin against marine biofilms was discovered during screening. Seven out of ten strains of bacteria isolated from marine biofilms had successfully formed biofilms under the testing situation, and elasnin showed great inhibiting efficiency against all biofilm-forming strains. The inhibiting efficiency of elasnin-based coatings against natural marine biofilms was then validated in the field test, which indicated a great predictive validity of our screening assays.

Elasnin was first discovered in 1978 as a new elastase inhibitor with low toxicity in mice and high selectivity for human granulocyte elastase [23], and its antimicrobial and antibiofilm activities have never been discovered. Based on the test results in the present study, elasnin not only inhibited the biofilm formation of both mono- and multispecies biofilms but also inhibited the settlement of large biofouling organisms. The 16S amplicon analysis revealed that the species richness and diversity of biofilms on the elasnin-based coatings were reduced, which might indirectly inhibit the settlement of biofouling organisms because the changed microbial compositions of the biofilms might not be suitable for them to settle on. In addition, the antilarval-settlement assay showed that elasnin could inhibit larval settlement, which had low toxicity. Considering the effective concentration of elasnin and the water mobility in the marine environment, the toxic effect of elasnin should be negligible, although a high mortality rate was recorded under the high concentration after 48 h exposure. However, the present study only superficially assessed the potential toxic effect of elasnin by the mortality rate of the larva, and its environmental impact should be further explored.

In addition, the wide-type strain *S. mobaraensis* DSM 40847 produced elasnin in substantial quantity (0.33 g/L), which was considered as a new industrial-producing strain of antibiofilm agents. Low product yield has always been a limitation for the development and commercial applications of newly discovered biologically active compounds. The fermentation of this bacterium would address the supply problem that often limited natural products from biotechnical development, leading to the late-stage development of elasnin. Subsequently, elasnin-based coatings were easily prepared with low expenditures. During a 4 week observation, the coatings (10 wt%) inhibited the biofilm formation in the first 2 weeks and the larval settlement in the last 2 weeks. Coatings began to lose their effectiveness after the third week in the field probably because of the reduced release rate of elasnin. Since 2008, when tributyltin (TBT) was restricted by the implementation of the International Maritime Organization Treaty on biocides, the development of efficient and environmentally friendly surface coatings became a hot topic. Collectively, originating from nature, elasnin showed great antibiofilm and antifouling activities with low toxic effects; combined with the low cost of supply, elasnin could provide a new selection for the development of antibiofilm and antifouling materials.

In the present study, we identified a potent antibiofilm compound, namely, elasnin, from the strain *S. mobaraensis* DSM 40847 in the course of our screening program using bacteria isolated from marine biofilms. With high productivity, elasnin-based antibiofilm coatings were easily prepared, which presented a favorable performance in inhibiting the biofilm formation and attachment of macro-foulers in the marine environment. The 16S amplicon and antilarval settlement assays revealed that the antifouling performance of elasnin-based coatings might be caused by the indirect effect of elasnin on biofilm’s microbial compositions and its direct inhibitory effect on the larval settlement. With low toxicity, high efficiency, and high productivity, elasnin showed great potential in the applications of biofilm and biofouling control in the marine environment.

## 4. Materials and Methods

### 4.1. Strains, Culture Media, and Chemicals

*Streptomyces mobaraensis* DSM 40847 was purchased from the German Collection of Microorganisms and Cell Cultures (DSMZ, Braunschweig, Germany). The marine bacteria *V. alginolyticus* B1, *Erythrobacter* sp. HKB8, *Ruegeria* B32, *S. aureus* B04, *S. hominis* N32, *S. arlettae* OM, *M. esteraromaticum* N22, *I. sediminum* N28, *Pseudoalteromonas* L001, and *E. coli* N57 were isolated from marine biofilms and obtained from the culture collection of our laboratory [24]. Soybean powder was purchased from Wugumf, Shenzhen, China. Soluble starch was purchased from Affymetrix, Santa Clara, CA, USA. Magnesium sulfate hydrate was purchased from Riedel-de-Haën, Seelze, Germany. Bacteriological peptone was obtained from Oxoid, Milan, Italy. Mueller-Hinton broth (MHB) was purchased from Fluka Chemie AG, Buchs, Switzerland. Phosphate-buffered saline (PBS) and 3-(4,5-dimethylthiazol-2-yl)-2,5-diphenyltetrazolium bromide (MTT) were purchased from Thermo Fisher Scientific Inc., San Jose, CA, USA. Lysogeny broth (LB), glucose, and 1-butanol were purchased from VWR International Ltd., Leicestershire, UK. All other chemicals were supplied by Sigma-Aldrich Corporation, Saint Louis, MO, USA.

### 4.2. Bioactive Compound Isolation and Identification

Stock cultures of *S. mobaraensis* DSM 40847 were inoculated into 50 mL of AM4, AM5, and AM6 media (Table S1) containing glass beads (to break up globular colonies) and incubated at 30 °C on a rotary shaker (170 rpm). The culture broth was extracted with 1-butanol on days 3, 5, and 7. The crude extracts were dissolved in DMSO before storage and bioassay. Pure compounds were isolated by reversed-phase HPLC (Waters 2695, Milford, MA, USA) using a semi-prep C18 column (10 × 250 mm) that was eluted with a 55 min gradient of 5–95% aqueous acetonitrile containing 0.05% trifluoroacetic acid at a flow rate of 3 mL/min. The structure of elasnin was elucidated through NMR analysis of ^1^H, ^1^H-^1^H-COSY, ^1^H-^13^C-HSQC, and ^1^H-^13^C-HMBC NMR spectra recorded on a Bruker AV500 NMR spectrometer (Bruker, Billerica, MA, USA) and ^13^C-NMR spectra obtained with the Bruker DRX600 NMR Spectrometer (Bruker, Billerica, MA, USA) using dimethyl sulfoxide-d_6_ (^1^H-NMR DMSO-d_6_: δH = 2.50 ppm; DMSO-d_6_: δC = 39.50 ppm).

### 4.3. Productivity Monitoring and Extraction Efficiency Comparison

A stock culture of *S. mobaraensis* DSM 40847 was incubated in the AM4 medium. One milliliter of culture broth was collected every 12 h and extracted with 1 mL of 1-butanol, ethyl acetate, or n-hexane. The solvent for extraction was then removed by evaporation. The crude extract was dissolved in methanol and quantified through HPLC analysis with a Phenomenex Luna C18 column. The peak of elasnin was identified from the retention time, and its concentration was calculated on the basis of an established standard curve (Figure S9).

### 4.4. MBIC Assay and MIC Assay Against Marine Bacteria

MBICs were determined as previously described [25,26]. In brief, an overnight culture of test strains was diluted into approximately 10^7^ CFU/mL with LB and 0.5% glucose and treated with various concentrations of elasnin (or only media for control) in 96-well cell culture plates. Then, the plates were incubated at 37 °C for 24 h and rinsed two times with 1 × PBS to remove non-adhering and planktonic cells. After rinsing, an MTT staining assay was conducted to measure viable cells in the biofilms because MTT could react with activated succinate dehydrogenase in the mitochondria of viable cells to form blue-violet formazan, which could be read at 570 nm after dissolving in DMSO. The MBIC_50_ and MBIC_90_ were defined as the lowest concentration needed for inhibiting 50% and 90% of biofilm formation individually. The biofilm inhibition efficiency was calculated using the following equation: Biofilm inhibition (%) = (OD_570nm_ of test compound) / (OD_570nm_ of control) × 100%. The experiments were performed in triplicate and repeated three times.

MICs were determined with test strains according to the Clinical and Laboratory Standards Institute guideline CLSI M100 (2018). Briefly, a 10^5^ CFU/mL overnight culture of test strains was inoculated into MHB and treated with elasnin (or only media for control) at a series of concentrations. After incubation for 24 h, the minimum concentrations at which no bacterial growth was visible were recorded as the MICs. The experiments were performed in triplicate and repeated twice; vancomycin and kanamycin were used as a positive control in the experiments.

### 4.5. Elasnin-Based Antibiofilm Coating Preparation

A 4 L culture broth of *S. mobaraensis* DSM 40847 (incubated as previously described) was extracted using n-hexane to obtain a sufficient amount of high-elasnin-content crude extracts. The elasnin-based antibiofilm coatings were prepared following the same procedures as those described by Ma et al. (2017). For the 10 wt% coatings, polymer (0.90 g, 90 wt%) and crude extracts (0.10 g, 10 wt%) were dissolved by vigorously stirring xylene and tetrahydrofuran (v:v = 1:2) at 25 °C. After mixing, a glass slide was coated with the solution and left to dry at room temperature for a week to remove the solvent. The same procedure was used in preparing coatings with different concentrations of crude extracts.

### 4.6. Field Test and Release Rate Determination

Coated glass slides were submerged in seawater at a fish farm in Yung Shue O, Hong Kong (114°21′ E, 22°24′ N) for 2 to 4 weeks. Afterward, the glass slides were retrieved and transported back to the laboratory in a cooler with in situ seawater and were washed two times using an autoclave and 0.22 μm filtered seawater (FSW) to remove loosely attached particles and cells. The slides were then stained using the FilmTracer™ LIVE⁄DEAD Biofilm Viability kit and investigated under CLSM (Zeiss LSM710, Carl Zeiss, Oberkochen, Germany). Moreover, the release rate of elasnin was determined by measuring its concentration using HPLC under static conditions. The coated panels were immersed in 100 mL of sterilized artificial seawater held in a measuring container. Ten milliliters of seawater was collected after immersion for 24 h, and elasnin was extracted with the same volume of dichloromethane, which was then removed under nitrogen gas. After drying, the extract was resuspended in 100 mL of methanol and underwent HPLC analysis. The release rate was measured every week for 4 straight weeks, and each concentration was tested in duplicate.

### 4.7. DNA Extraction, 16S rRNA Gene Sequencing, and Analyses

Biofilm samples on the coated slide surface were collected with autoclaved cotton and stored in DNA storage buffer (10 mM Tris-HCl; 0.5 mM EDTA, pH 8.0) at −80 °C. Before the extraction, samples were vortexed several times to release the microbial cells into the DNA storage buffer. All the samples were then subjected to centrifugation at 10,000 rpm for 1 min, and the supernatant was discarded. After continuous treatment with 10 mg/mL of lysozyme and 20 mg/mL of proteinase K, DNA was extracted from the treated microbial cells with a microbial genomic DNA extraction kit (Tiangen Biotech, Beijing, China) following the manufacturer’s protocol.

The quality of DNA samples was controlled using NanoDrop (which tested DNA purity, OD260/OD280) and agarose gel electrophoresis (which tested DNA degradation and potential contamination). The hypervariable V3-V4 region (forward primer: 5′-CCTAYGGGRBGCASCAG-3′; reverse primer: 5′-GGACTACNNGGGTATCTAAT-3′) of prokaryotic 16S rRNA genes was used to amplify DNA from biofilms by polymerase chain reaction (PCR). The PCR products were purified before library construction and sequenced at Novogene (Beijing, China) on the NovaSeq 6000 System. The read length was 250 bp, and each pair of reads had a 50 bp overlapping region. The paired-end reads were subjected to quality control using the NGS QC Toolkit (version 2.0, The National Institute of Plant Genome Research, New Delhi, India) [27]. The 16S rRNA gene amplicon data were analyzed using the software package QIIME2 and then merged using Q2_manifest_maker.py in QIIME2 [28]. The low-quality reads and chimeras were removed using dada2 commands in QIIME2. A total of 500,000 filtered reads for each sample were selected to normalize the uneven sequencing depth. OTUs were classified de novo from the pooled reads at 97% sequence similarity using a classifier trained by the Naive Bayes method. Representative sequences were then recovered using the feature-classifier classify-sklearn script in QIIME2. The alpha-diversity analyses (observed OTUs and Shannon diversity) were performed using the script “qiime diversity alpha” in QIIME2. Beta-diversity based on the Bray–Curtis distances was conducted by the cluster analysis in the software PAST (version 3.0) [29]. Furthermore, the taxonomic structure was drawn in Excel wo (Office 365 MSO 64-bit) on the basis of the relative abundance.

### 4.8. Antilarval-Settlement Assay

The direct antilarval-settlement assay was conducted using cyprids of the barnacle *B. amphitrite* Darwin as described previously [30,31,32,33]. In brief, adult *B. amphitrite* (Darwin) were collected from the intertidal zone in Pak Sha Wan, Hong Kong (22°19′ N, 114°16′ E) and raised to competence for experiments. Elasnin was dissolved in DMSO and diluted into four concentrations from 50 to 6.25 μg/mL with a twofold serial dilution. DMSO was used as a negative control. About 10 ± 2 *B. amphitrite* cyprids were inoculated into each well of a 24-well polystyrene culture plate that contained 2 mL of 0.22 μm FSW with different treatments. For all treatments and controls, three replicates were performed. The plates were then incubated at 25 °C in darkness. After 24 and 48 h, the number of settled and swimming larvae was counted using a Leica MZ6 microscope (Leica Microsystems, Wetzlar, Germany), and possible toxic effects were also noted.

### 4.9. Statistical Analyses

Statistical analyses for all data were performed using the GraphPad Prism 8.0.2 software (San Diego, CA, USA). The composition of the biofilm on the coatings was compared with that in the control groups using Student’s t-test.

## 5. Patents

The authors declare the following competing interests: This work has been submitted for the U.S. Patent Application (No. 16999437) and Chinese Patent application (No. 202010850564.X).

## Data Availability

The data presented in this study are available in the main text and the supplementary materials of this article.

## Supplementary Materials

The following are available online at https://www.mdpi.com/1660-3397/19/1/19/s1, Table S1: Media used in this study, Figure S1: Bioactivities of crude extract of the secondary metabolites produced by *S. mobaraensis* DSM 40847 (incubated with AM4 media and extracted with 1-butanol) and 20 fractions of it. Fraction 17 and 15 are the analogs of franction 16 (elasnin), Figure S2: ^13^C-NMR analysis of bioactive fraction 16 (Elasnin), Figure S3: ^1^H-NMR analysis of bioactive fraction 16 (Elasnin), Figure S4: ^1^H-^1^H COSY of bioactive fraction 16 (Elasnin), Figure S5: ^1^H-^13^C HSQC of bioactive fraction 16 (Elasnin), Figure S6: ^1^H-^13^C HMBC of bioactive fraction 16 (Elasnin), Table S2 ^13^C-NMR (150 MHz, DMSO-d_6_), ^1^H-NMR (500 MHz, DMSO-d_6_) and HMBC correlations of compound Fraction 16 (DMSO-d_6_) and comparisons between Elasnin (Omura, Nakagawa et al. 1979), Figure S7: HPLC profile of high-elasnin-content crude extracts and productivity of crude extracts/elasnin by using different extraction solvent, Figure S8: Microbial compositions of biofilms on control slides (C-1,2,3) and 10 wt% elasnin-based (E10-1,2,3) coatings at the genus level, Figure S9: Stand curve of elasnin acquired by HPLC.
