# Supplementary material for: Discovery of Antibiofilm Activity of Elasnin against Marine Biofilms and Its Application in the Marine Antifouling Coatings
Source: Mar Drugs. 2021 Jan 5;19(1):19. doi: 10.3390/md19010019 (PMC7824865; doi:10.3390/md19010019)
Supplement: Supplementary file 1 [file marinedrugs-19-00019-s001.pdf]

Table S1. Media used in this study.

Media	Formula
AM4	Soybean Powder 20 g/L, Peptone Bacteriological 2 g/L, Glucose 20 g/L, Soluble starch 5 g/L, Yeast Extract 2 g/L, NaCl 4 g/L, K_2HPO_4 0.5 g/L, $MgSO_4 \cdot 7H_2O$ 0.5 g/L, $CaCO_3$ 2 g/L, pH 7.8
AM5 (ISP2)	Malt extract 10 g/L, Yeast extract 4 g/L, Glucose 4 g/L
AM6	Soluble starch 20 g/L, Glucose 10 g/L, Peptone Bacteriological 5 g/L, Yeast extracts 5 g/L, $CaC0_3$ 5 g/L, pH 7.2-7.5
GYM	Glucose 4.0 g, Yeast extract 4.0 g, Malt extract 10.0 g, CaCO3 2.0 g, Agar 12.0 g, Distilled water 1000.0 ml (Adjust pH to 7.2 before adding agar.)

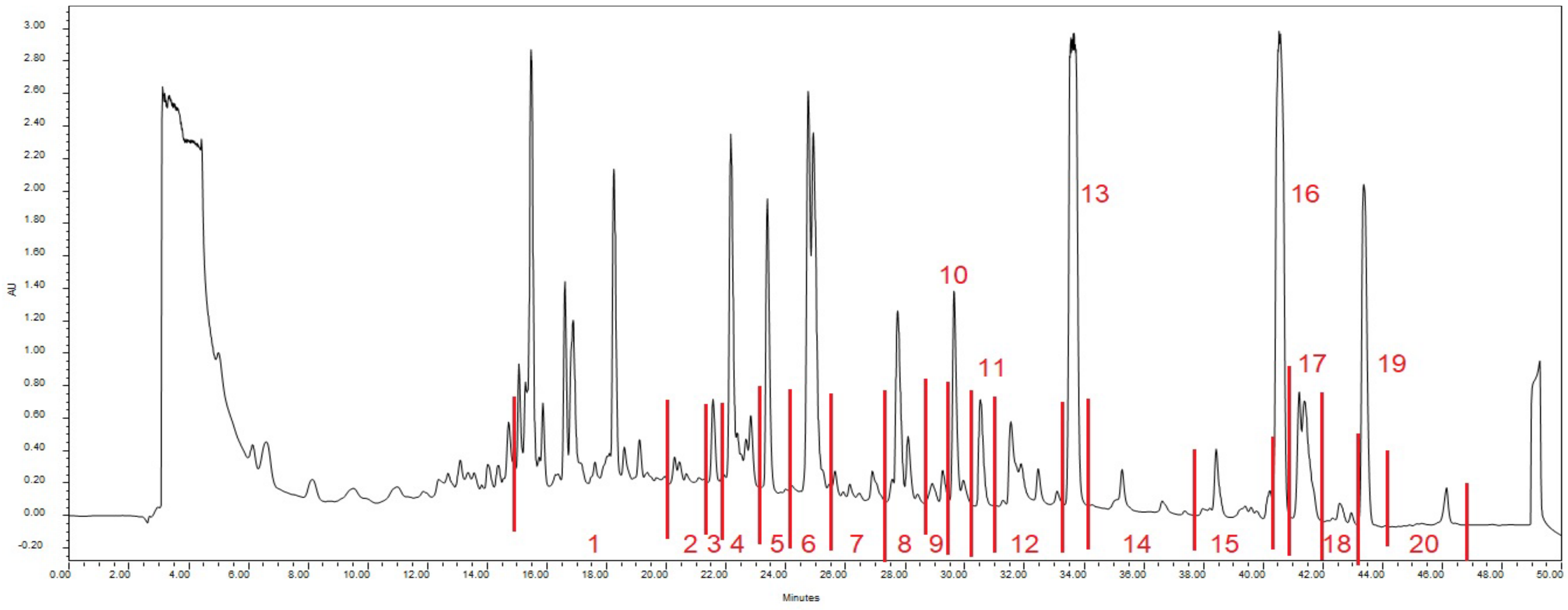

Concentration(μg/ml)	MIC	MBIC
>100	1-14; 18-20	1-15; 18-20
20-100	15	17 and 15
4-20	17	-
<4	16 and crude extracts	16 and crude extracts

Figure S1. Bioactivities of crude extract of the secondary metabolites produced by *Streptomyces mobaraensis* DSM 40847 (incubated with AM4 media and extracted with 1-butanol) and 20 fractions of it. Fraction 15 and 17 are the analogs of fraction 16 (elasnin).

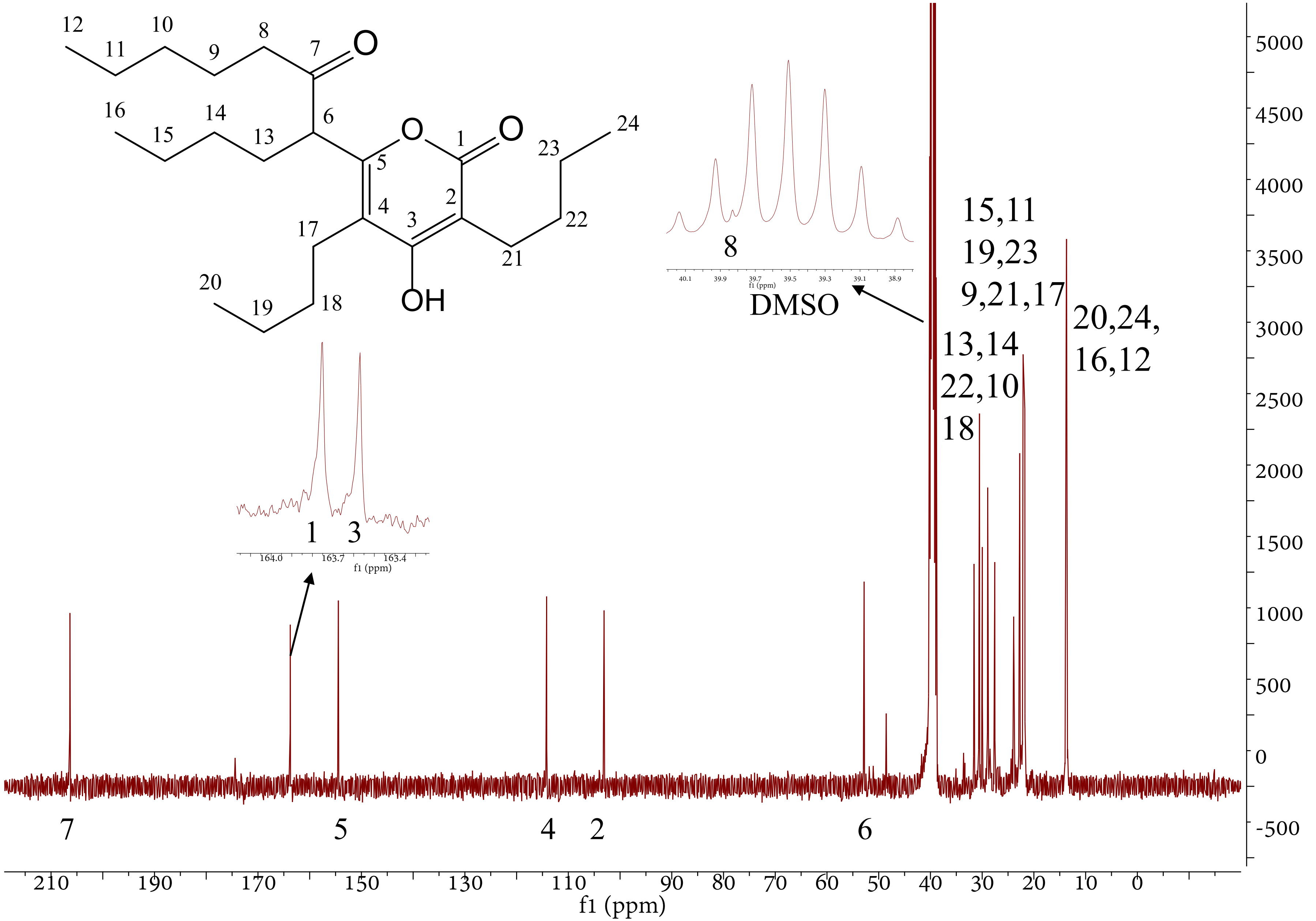

Figure S2. ^{13}C-NMR analysis of bioactive fraction 16 (Elasnin).

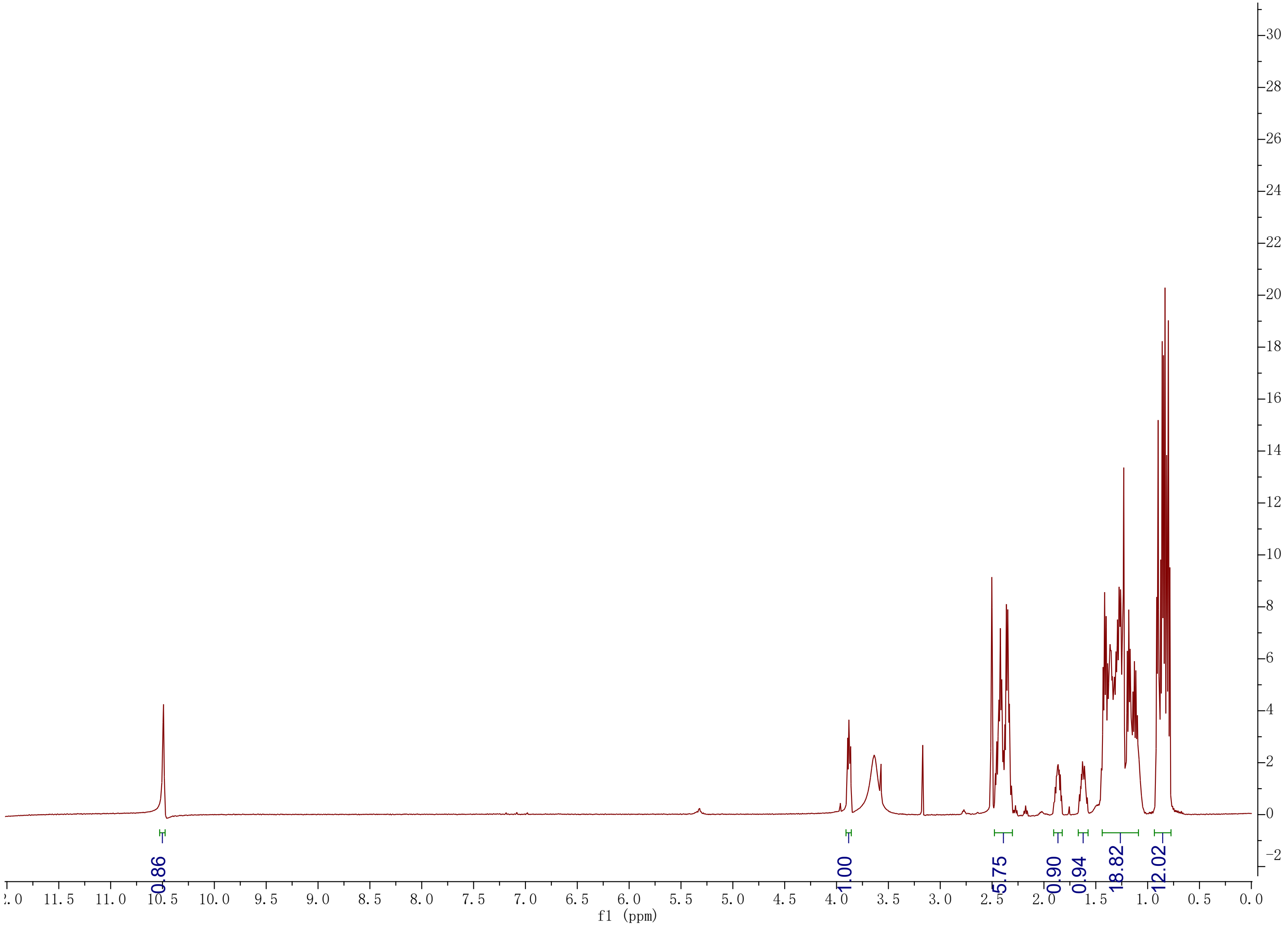

Chemical Shift (ppm)	Multiplicity	Coupling Constant (Hz)	Integration
10.49	s	–	1H
3.87	dd	8.8, 5.7	1H
2.32-2.47	m	–	6H
1.86	m	–	1H
1.61	m	–	1H
1.04-1.45	overlapped	–	18H
0.90	t	–	3H
0.86	t	–	3H
0.83	t	–	3H
0.80	t	–	3H

Figure S3. ^{1}H-NMR analysis of bioactive fraction 16 (Elasnin).

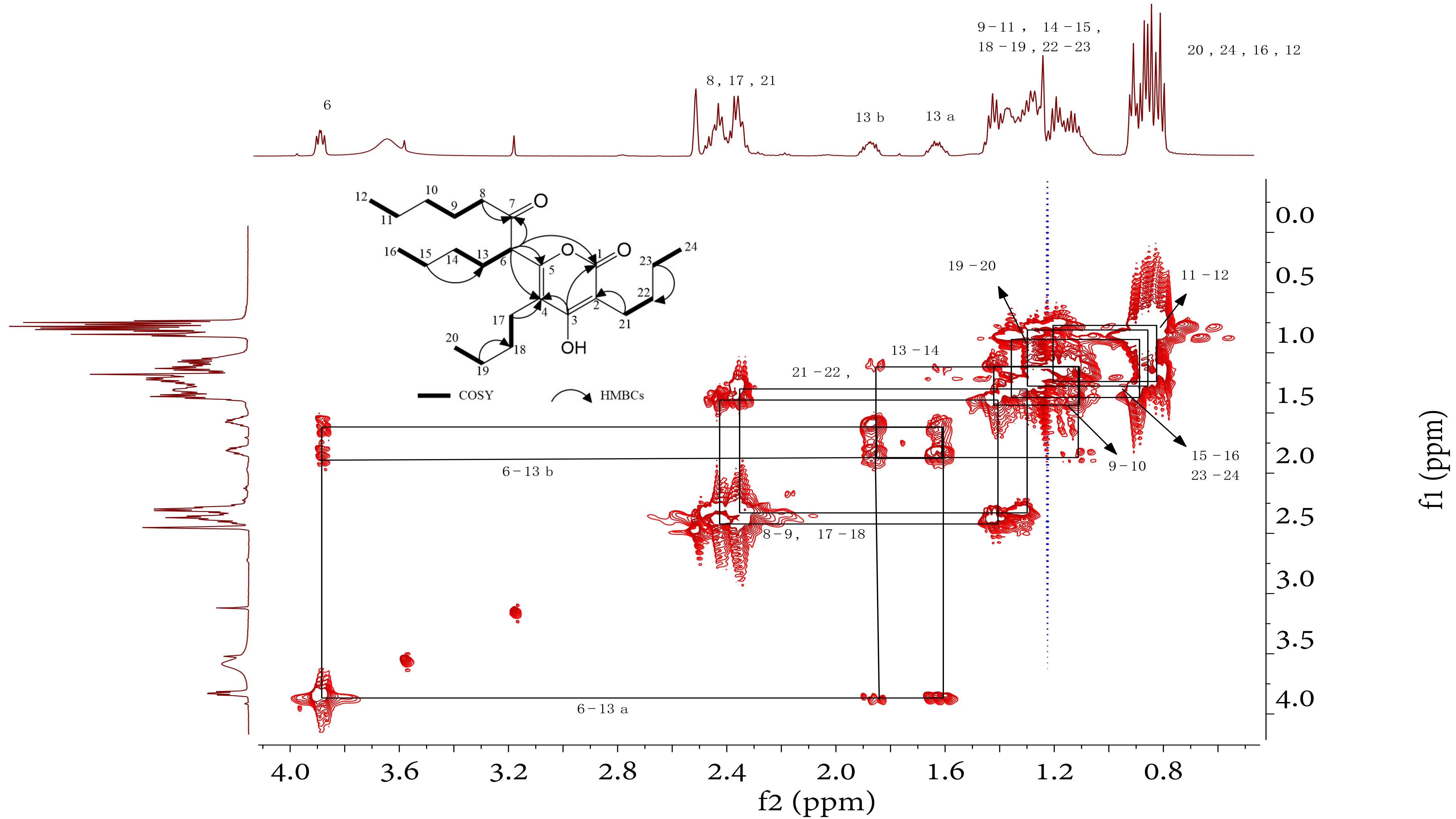

Figure S4. ¹H-¹H COSY of bioactive fraction 16 (Elasnin).

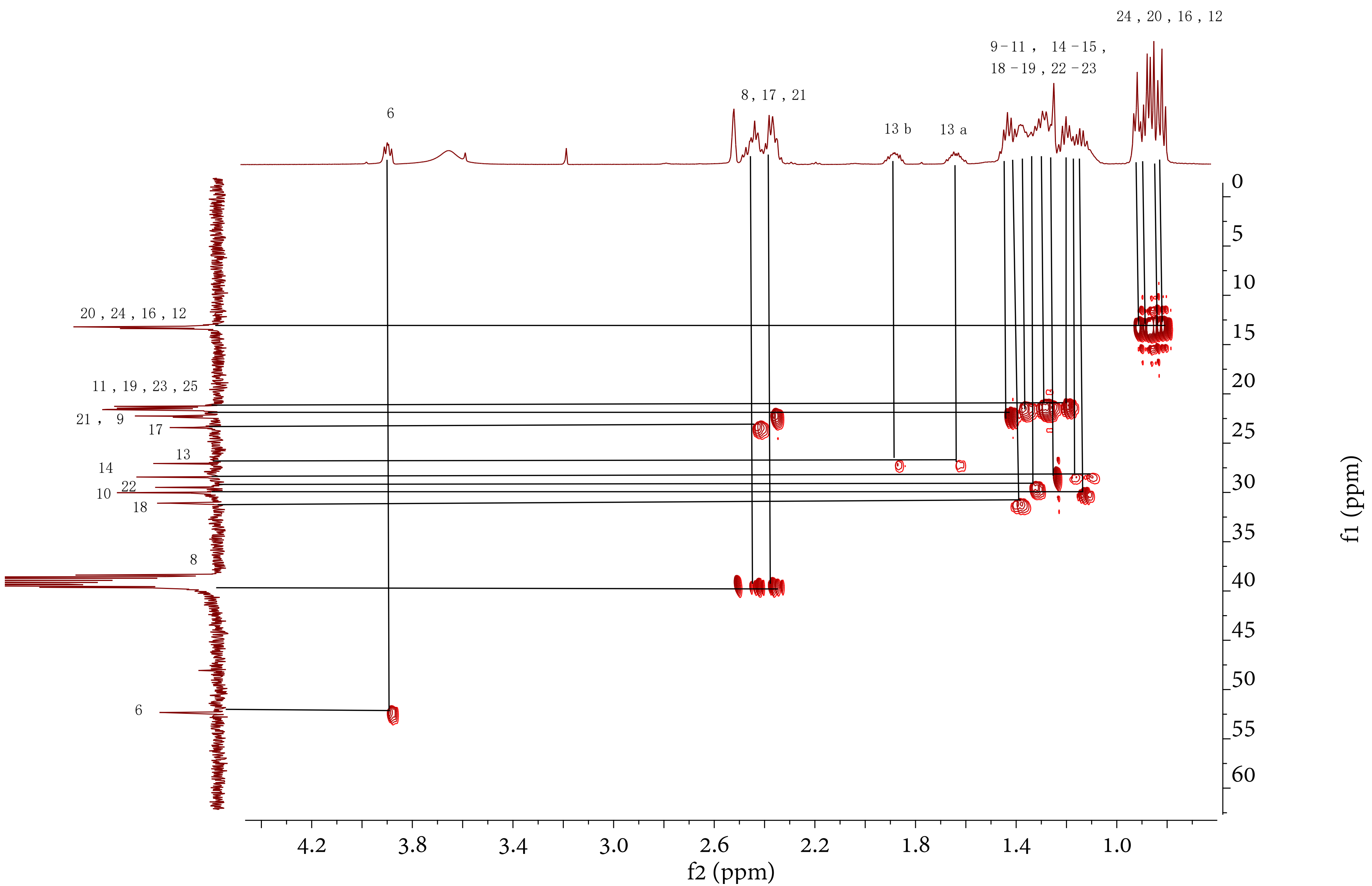

Figure S5. ^{1}H-^{13}C HSQC of bioactive fraction 16 (Elasnin).

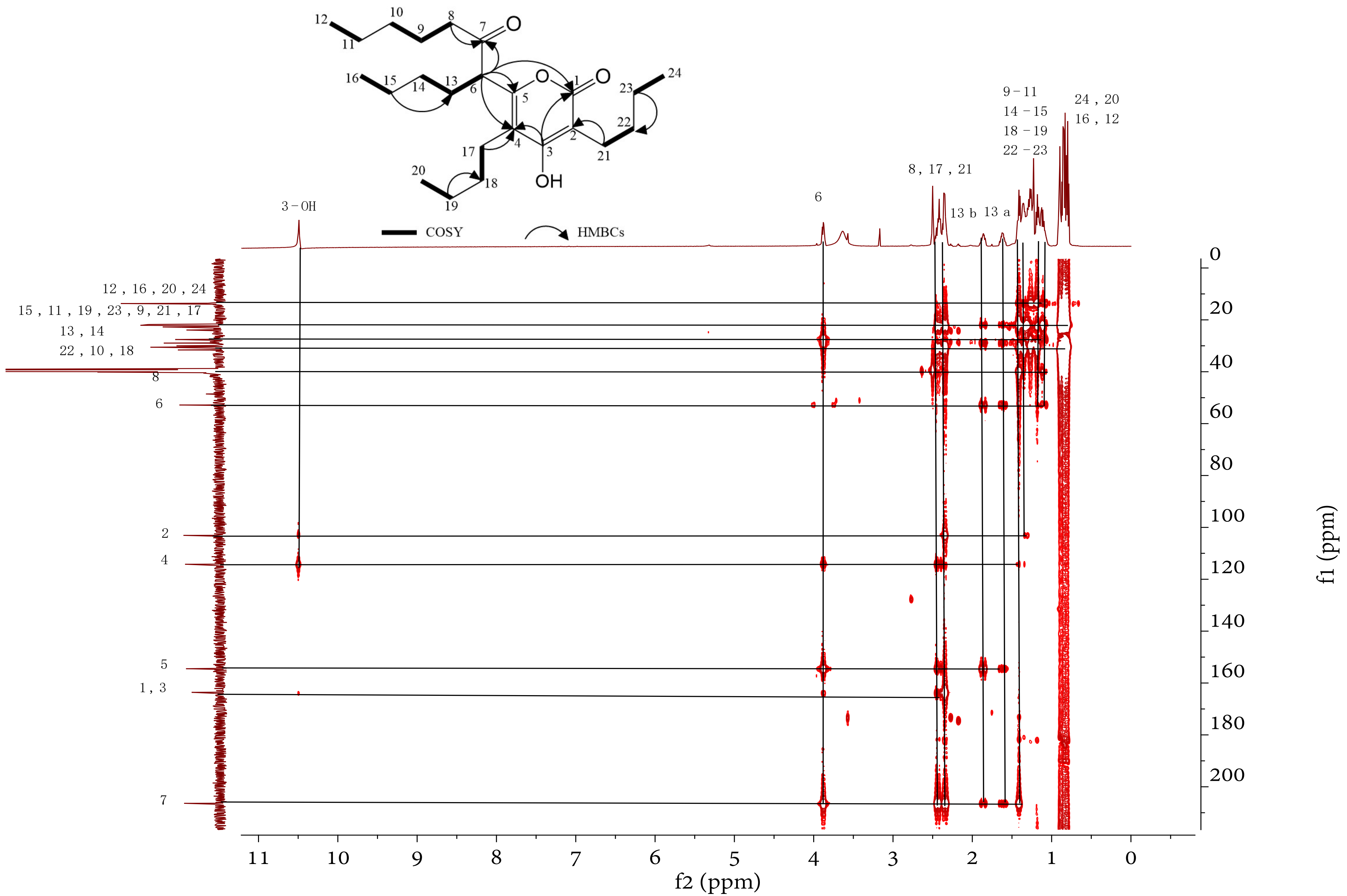

Figure S6. ^{1}H-^{13}C HMBC of bioactive fraction 16 (Elasnin).

Table S2. ^{13}C-NMR (150 MHz, DMSO-d_6), ^{1}H-NMR (500 MHz, DMSO-d_6) and HMBC correlations of compound Fraction 16 (DMSO-d_6) and comparisons between Elasnin (Omura, Nakagawa et al. 1979).

Position	Fraction 16			Elasnin
	δC (mult.)	δH [mult., J (Hz)]	HMBC (H→C#)	δC
1	163.8 (qC)		OH-3, H-6, H_2-21	165.5
2	103.1 (qC)		OH-3, H_2-21, H_2-22, H_2-23	104.3
3	163.6 (qC)		H_2-17	164.5
3-OH		10.49 (s)		
4	114.2 (qC)		OH-3, H-6, H_2-17, H_2-18	114.9
5	154.5 (qC)		H-6, H_2-8, H_2-13, H_2-17	154.8
6	52.8 (CH)	3.87 (dd, 8.8, 5.7)	H_2-13	54.7
7	206.8 (qC)		H-6, H_2-8, H_2-9, H_2-13	206.9
8	39.8 (CH_2)	2.35 (m), 2.42 (m)	H_2-9, H_2-10	40.2
9	22.8 (CH_2)	1.42 (m)	H_2-8, H_2-11	
10	30.5 (CH_2)	1.11 (m)	H_2-8, H_2-9, H_2-11, H_2-12	22.4-40.3
11	22.1 (CH_2)	1.19 (m)	H_2-12	
12	13.8 (CH_3)	0.80 (t, 5.0)	H_2-10, H_2-11	13.9 (CH_3)
13	27.6 (CH_2)	1.61 (m), 1.86 (m)	H-6, H_2-15	
14	28.9 (CH_2)	1.23 (m)	H_2-16	22.4-40.3
15	22.0 (CH_2)	1.27 (m)	H_2-16	
16	13.8 (CH_3)	0.83 (t, 5.0)	H_2-14, H_2-15	13.9 (CH_3)
17	23.9 (CH_2)	2.43 (m)		
18	31.5 (CH_2)	1.41 (m)	H_2-19, H_2-20	22.4-40.3
19	22.1 (CH_2)	1.35 (m)	H_2-17, H_2-18, H_2-20	
20	13.8 (CH_3)	0.90 (t, 5.0)	H_2-19	13.9 (CH_3)
21	22.8 (CH_2)	2.34 (m)	H_2-22	
22	30.0 (CH_2)	1.31 (m)	H_2-23, H_2-24	22.4-40.3
23	22.1 (CH_2)	1.27 (m)	H_2-21, H_2-22, H_2-24	
24	13.8 (CH_3)	0.86 (t, 5.0)	H_2-23	13.9 (CH_3)

Extraction solvent-Time	Elasnin (mg/L)	crude extracts (mg/L)	Elasnin/Crude Extract(%)
Hexane-2d	216.02	533.33	40.50
Butanol-2d	680.78	3866.67	17.61
EA-2d	759.96	2400.00	31.67
Hexane-4d	228.71	800.00	28.59
Butanol-4d	285.58	3600.00	7.93
EA-4d	430.50	1333.33	32.29
Hexane-6d	336.63	1733.33	19.42
Butanol-6d	147.37	3600.00	4.09
EA-6d	62.91	666.67	9.44

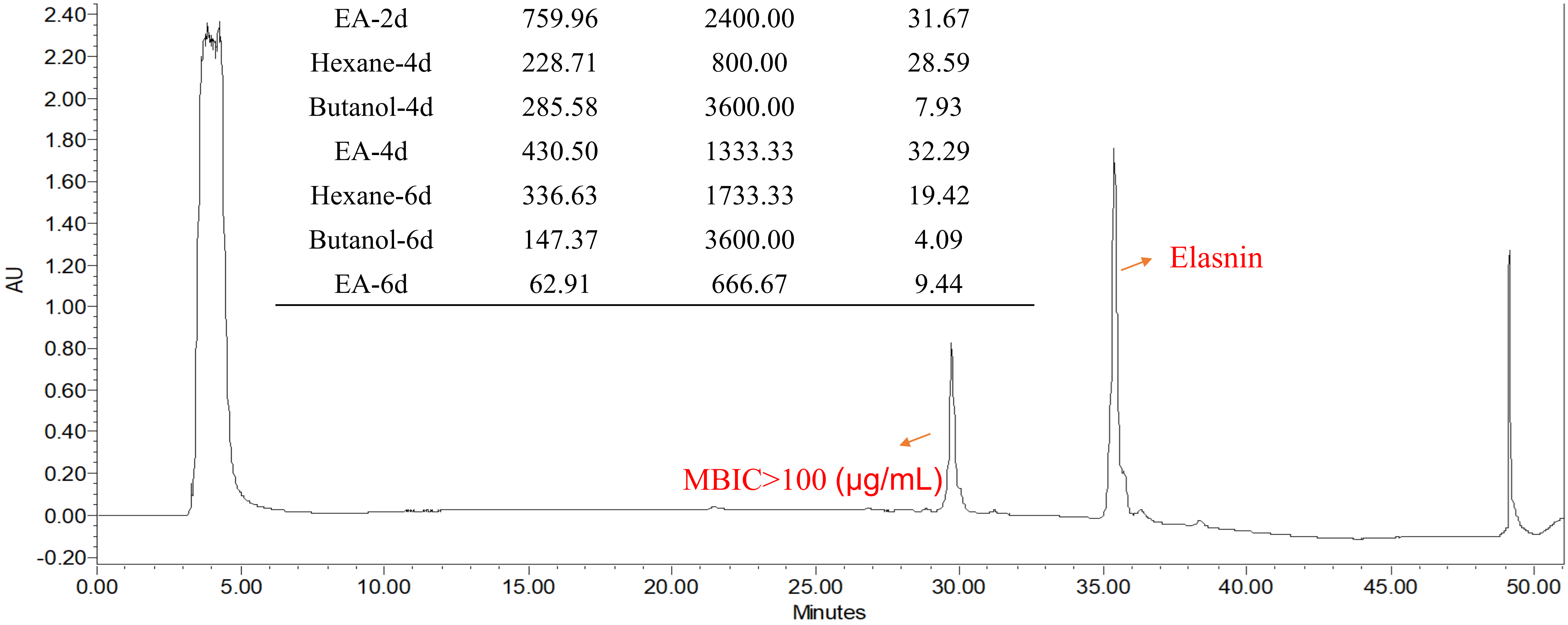

Figure S7. HPLC profile of high-elasnin-content crude extracts and productivity of crude extracts/elasnin by using different extraction solvent.

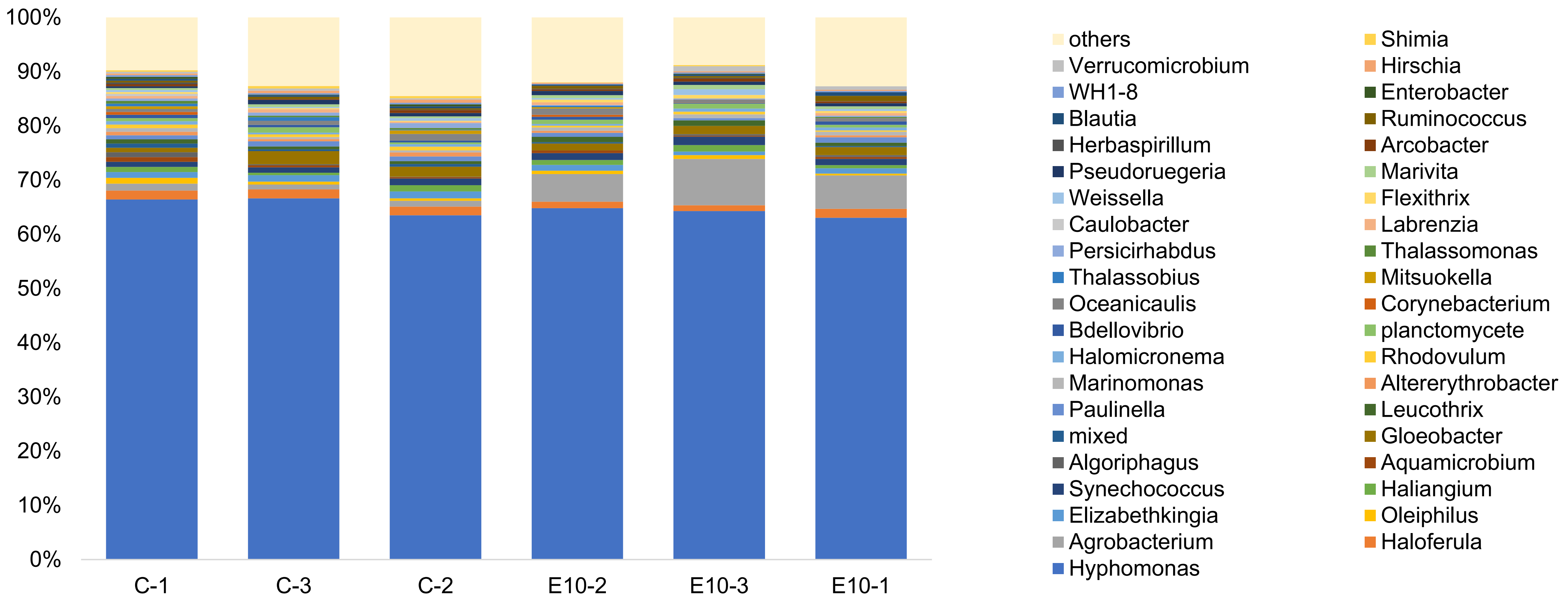

Figure S8. Microbial compositions of biofilms on control slides (C-1,2,3) and 10 wt% elasnin-based (E10-1,2,3) coatings at the genus level.

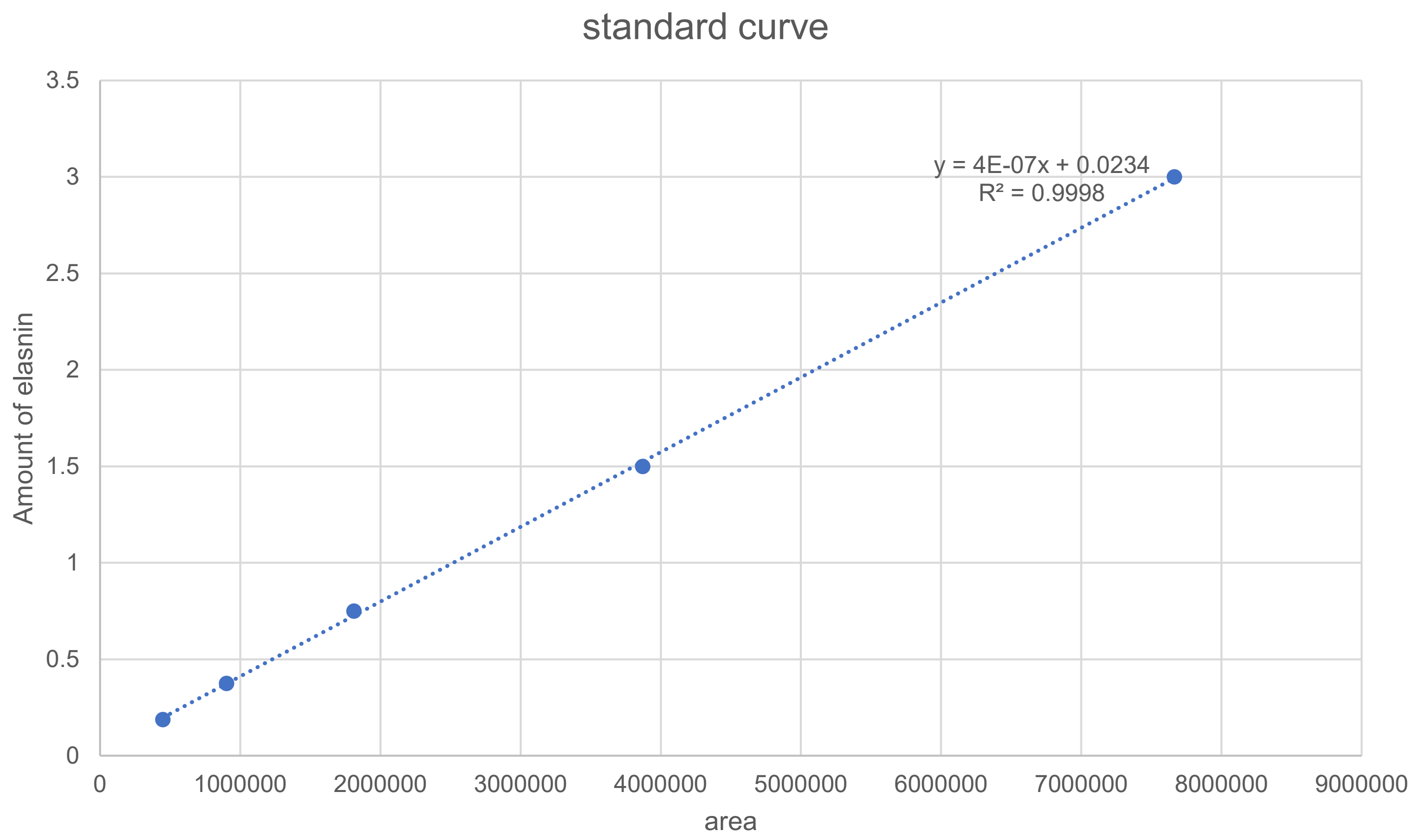

Concentration (mg/ml)	dilution	injection(μl)	area	amount （μg)
10	100	30	7663013	3
10	200	30	3870276	1.5
10	400	30	1811181	0.75
10	800	30	902887	0.375
10	1600	30	446899	0.1875

Figure S9. stand curve of elasnin acquired by HPLC.
